# Too little or too much corticosteroid? Coexisting adrenal insufficiency and Cushing's syndrome from chronic, intermittent use of intranasal betamethasone

**DOI:** 10.1530/EDM-13-0036

**Published:** 2013-10-04

**Authors:** Adrienne Dow, Run Yu, John Carmichael

**Affiliations:** 1 Division of EndocrinologyCedars-Sinai Medical Center8700 Beverly Blvd, B-131, Los Angeles, California, 90048USA

## Abstract

To report the puzzling, rare occurrence of coexisting adrenal insufficiency and Cushing's syndrome from chronic, intermittent use of intranasal betamethasone spray. A 62-year-old male was referred to our endocrinology clinic for management of adrenal insufficiency. This previously healthy individual began to experience chronic sinus symptoms in 2007, was treated with multiple ensuing sinus surgeries, and received oral glucocorticoid for 6 months. In the following 5 years, he suffered severe fatigue and was diagnosed with secondary adrenal insufficiency. He could not be weaned from corticosteroid and developed clear cushingoid features. In our clinic, careful inquiry on medications revealed chronic, intermittent use of high-dose intranasal betamethasone since 2008, which was not apparent to his other treating physicians. His cushingoid features significantly improved after holding intranasal betamethasone.

**Learning points:**

- Chronic, intermittent intranasal betamethasone can cause secondary adrenal insufficiency and iatrogenic Cushing's syndrome when used in excess.
- Topical corticosteroid use should be considered in the differential diagnosis of adrenal insufficiency or Cushing's syndrome.

## Background

Topical corticosteroids have long been used for the treatment of allergic rhinitis and chronic sinusitis with satisfactory symptomatic relief (1). They are generally regarded as safe due to its local effects and limited systemic absorption. We report here a case of coexisting secondary adrenal insufficiency and iatrogenic Cushing's syndrome from chronic, intermittent intranasal corticosteroid use.

## Case presentation

A 62-year-old male was referred to our endocrinology clinic for adrenal insufficiency management in 2013. The patient had been in excellent health and had run seven marathons until 2007 when his chronic sinusitis became more severe. In that year, he underwent sinus surgery with removal of polyps. Prior to surgery and after surgery, the patient received prednisone with a maximum dose of 30 mg with gradual taper over 6 months. The patient subsequently began to experience fatigue and increased sinus congestion with multiple sinus infections. By the fall of 2009, his general health condition began to decline. The patient underwent a second sinus surgery for his symptoms. In 2010, his energy level decreased to the effect that he could no longer train for marathons nor run his previous average of 40 miles/week. He was seen by an outside endocrinologist in 2010 and secondary adrenal insufficiency was suspected. His morning cortisol levels were extremely low at 0.2 μg/dl (normal reference range 8–20.0 μg/dl) and adrenocorticotropin (ACTH) <1.0 pg/ml (normal reference range 7.2–63.3 pg/ml). An ACTH stimulation test revealed suppressed basal cortisol levels (0.3 μg/dl) and only 3.3 and 4.6 μg/dl at 30 and 60 min respectively (normal reference range after ACTH >20.0). His prolactin levels were normal but total testosterone levels were slightly low at 2.5 ng/ml (normal reference range 2.8–8.0 ng/ml). Magnetic resonance imaging (MRI) of the brain with and without contrast demonstrated normal brain but chronic sinusitis in the sphenoid sinuses and right maxillary sinus. Dedicated pituitary MRI was not performed. The patient was diagnosed with secondary adrenal insufficiency of unclear etiology. Hydrocortisone was initiated, but the patient continued to feel fatigue with usual doses and initially required 30 mg hydrocortisone in divided doses daily to improve energy levels. The patient subsequently gained 15 pounds, developed truncal obesity, buffalo hump, hypertension, easy bruisability, low libido, and proximal muscle wasting. In 2011, the patient continued to have suppressed cortisol levels of <0.5 μg/dl and ACTH of <5 pg/ml. The patient noted decreased vision in both eyes in 2009 and was treated by an ophthalmologist in 2010 with subsequent cataract surgery in the middle of 2011. Several months later, the patient was admitted for septic shock without severe abdominal pain or fever and was diagnosed with a ruptured diverticular abscess with peritonitis, for which he underwent low anterior abdominal resection. Later in 2011, the patient was reportedly osteopenic. In 2012, the patient had his third sinus surgery and later underwent anterior cervical fusion for degenerative disk disease with placement of spacers. Repeated attempts of corticosteroid dose tapering were met with severe fatigue and the patient continued to feel fatigued with usual doses of hydrocortisone (10 mg in the morning and 5 mg in the afternoon). Cortisol and ACTH levels remained suppressed and the cause of his secondary adrenal insufficiency is obscure.

## Investigation

**Figure 1:**
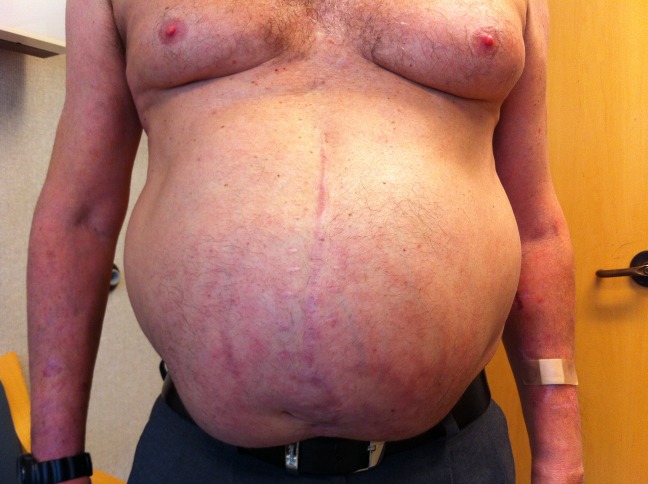
The patient's cushingoid abdomen with violaceous striae.

At our clinic, he was concerned at the failure of corticosteroid dose tapering and wondered why he seemed to have both adrenal insufficiency and Cushing's syndrome at the same time. He maintained that he did not take any corticosteroid other than the hydrocortisone given by the other endocrinologist after 2007, which was confirmed by his medication list. The patient additionally denied any prior corticosteroid injections. On physical examination, he was mildly overweight with BMI 25.8 kg/m^2^ (normal range of 20–25 kg/m^2^) but appeared clearly cushingoid. He exhibited facial swelling with plethora, dorsocervical dysplasia, gynecomastia, obese abdomen with violaceous lower abdominal striae (Fig. 1), muscle wasting of lower extremities, and diffuse ecchymoses. On further questioning of any potential nutrition supplements and topical medications, patient divulged that he had been using intermittent nasal betamethasone prescribed by his rhinologist since 2008. The dose of nasal betamethasone was initially 0.8 mg/ml, two sprays in each nostril every day (∼0.32 mg). For increased congestion, the patient would use, at most, four nasal sprays in each nostril (∼0.64 mg). The nasal betamethasone doses were escalated gradually due to persistent sinusitis. Most recently, he had used nasal betamethasone spray roughly 3.2 mg every other day. He also related that his energy levels were higher on the days he used betamethasone but would feel fatigued on the days he did not. Pituitary MRI done at our institution showed a relatively small but normal pituitary.

## Treatment

The patient was diagnosed with iatrogenic Cushing's syndrome secondary to use of nasal betamethasone with secondary adrenal insufficiency. He was asked to discontinue betamethasone as a diagnostic trial and to continue his current hydrocortisone dose of 10 mg in the morning and 5 mg in the afternoon.

## Outcome and follow-up

One week later, he exhibited visible improvement in facial swelling and plethora but reported fatigue, which was attributed to corticosteroid withdrawal. He was given hydrocortisone 20 mg in the morning and 10 mg in the afternoon with dose tapering. Two weeks after discontinuation of nasal betamethasone, he was on hydrocortisone 30 mg in divided doses daily with some fatigue; the dose is being tapered at the time of writing. His cushingoid features continued to diminish. Because of increased sinus congestion, the patient required the use of betamethasone twice. On those days, as instructed, he did not take hydrocortisone. 

**Figure 2:**
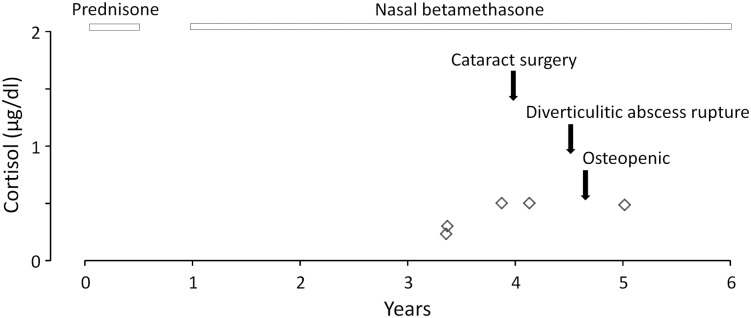
The patient's clinical course from initiation of systemic corticosteroid to presentation to our endocrinology clinic. The *y*-axis indicates morning plasma cortisol levels (note that the data are charted within the range from 0.0–4.0 μg/dl, with 4.0 μg/dl being the lower limit of normal among most laboratory values). The bold arrows indicate complications the patient faced from chronic steroid use.

The patient was followed up by his rhinologist, and given his uncontrolled symptoms, the patient and his rhinologist considered a potential fourth sinus surgery to relieve symptoms. At the time of writing, the patient's sinus symptoms appear stable without further sinus surgery. His overall clinical course is summarized in Fig. 2.

## Discussion

Steroid nasal preparations are commonly prescribed for the treatment of allergic rhinitis and non-allergic nasal diseases (1). Topical corticosteroid therapy for local disease is favored over systemic therapy to minimize systemic side effects and is often viewed as a benign medication with minimal side effects (1) (2). As such, both patients and physicians often do not consider topical corticosteroid therapy as a significant medication. In our patient, the cause of secondary adrenal insufficiency remained obscure until presentation to our clinic. Further inquiry led us to suspect nasal steroid spray as the culprit producing iatrogenic Cushing's syndrome with secondary adrenal insufficiency. 

The most common cause of secondary adrenal insufficiency is exogenous corticosteroid (3). Less frequently, pituitary and hypothalamic diseases can also cause secondary adrenal insufficiency. The normal findings on brain and pituitary MRI and the normal thyroid function tests and prolactin levels in our patient effectively ruled out pituitary or hypothalamic diseases as potential causes of the secondary adrenal insufficiency. While the systemic corticosteroid use in 2007 was suspected as the cause of the patient's secondary adrenal insufficiency, the persistently suppressed adrenal function is unusual, especially after the patient developed clear cushingoid features. The slightly higher doses of hydrocortisone for replacement (20 mg in the morning and 10 mg in the afternoon) are unlikely to cause overt Cushing's syndrome (3). There have been few reports describing Cushing's syndrome following the use of intranasal corticosteroids. Interestingly, previous case reports describe the use of intranasal betamethasone drops in comparison to betamethasone intranasal spray as we report here (4) (5) (6). In either case, topical betamethasone can lead to systemic absorption, via the nasal mucosa and via the gastrointestinal tract, through a significant amount swallowed. When swallowed, betamethasone undergoes a small degree of first pass hepatic metabolism and thereby remains systemically active in the bloodstream (7). Additionally, nasal absorption may be increased, depending on the extent of mucosal inflammation. Extensive inflammation can be presumed in this patient in light of his need for three sinus surgeries. Our patient began to experience extreme fatigue within a year of intranasal steroid initiation; 2 years later, extreme adrenal suppression was noted by laboratory testing. The highest dose taken roughly corresponded to an equivalent of 100 mg hydrocortisone every other day. Notably, the patient also developed cataracts and osteopenia, both known complications related to chronic steroid use. Furthermore, rupture of diverticular abscess in the presence of mild symptoms and lack of fever was likely reflective of the patient's suppressed immune status. While measurement of betamethasone levels in our patient was sought out, no such commercial laboratory test existed, offering a major limitation to our report. 

In summary, intranasal betamethasone spray can potentially lead to secondary adrenal insufficiency and iatrogenic Cushing's syndrome when used in excess. Careful inquiry as to the use of intranasal steroids should be pursued in all patients presenting with secondary adrenal insufficiency or Cushing's syndrome. Our case serves to highlight the importance of judicious use of topical preparations to prevent potentially serious adverse effects. It also highlights the need for thorough and careful histories taken by physicians investigating Cushing's syndrome and the need for adequate education for patients treated with all forms of steroid therapy.

## Patient consent

Patient has consented to the writing of this case report.

## Author contribution statement

All authors contributed to the writing of this paper. R Yu was the treating physician of the patient.
